# Roles and mechanisms of aberrant alternative splicing in melanoma — implications for targeted therapy and immunotherapy resistance

**DOI:** 10.1186/s12935-024-03280-x

**Published:** 2024-03-10

**Authors:** Wanxian Chen, Deyi Geng, Jiasheng Chen, Xiaosha Han, Qihu Xie, Genghong Guo, Xuefen Chen, Wancong Zhang, Shijie Tang, Xiaoping Zhong

**Affiliations:** 1 Department of Plastic and Burns Surgery, The Second Affiliated Hospital of Shantou University Medical College, Shantou, 515000 P. R. China; 2 Plastic Surgery Research Institute, Ear Deformities Treatment Center and Cleft Lip and Palate Treatment Center, Shantou University Medical College, Shantou, China

**Keywords:** Alternative splicing, Diagnostic biomarkers, Melanoma, Immunotherapy, Targeted therapy

## Abstract

**Background:**

Despite advances in therapeutic strategies, resistance to immunotherapy and the off-target effects of targeted therapy have significantly weakened the benefits for patients with melanoma.

**Main body:**

Alternative splicing plays a crucial role in transcriptional reprogramming during melanoma development. In particular, aberrant alternative splicing is involved in the efficacy of immunotherapy, targeted therapy, and melanoma metastasis. Abnormal expression of splicing factors and variants may serve as biomarkers or therapeutic targets for the diagnosis and prognosis of melanoma. Therefore, comprehensively integrating their roles and related mechanisms is essential. This review provides the first detailed summary of the splicing process in melanoma and the changes occurring in this pathway.

**Conclusion:**

The focus of this review is to provide strategies for developing novel diagnostic biomarkers and summarize their potential to alter resistance to targeted therapies and immunotherapy.

## Background

Melanoma is not as common as other skin cancers; however, it is more lethal, resulting in approximately 75% of skin cancer-related deaths [1]. The rapidly increasing incidence of melanoma and the high lethality of advanced disease have prompted efforts to identify factors that drive melanoma development and progression [2, 3]. The approval of several therapeutic agents against melanoma has revolutionized the treatment of patients with advanced‑stage disease [4]. Compared with a decade ago, the 5-year survival rate for patients with advanced melanoma treated with BRAF inhibitors, MEK inhibitors, or single PD-1 antibodies has improved from 5% to approximately 30% [5–7]. However, transcriptional reprogramming allows heterogeneous tumors to pass through different stages of melanoma progression and adapt to drug exposure during treatment [8–10], leading to targeted therapy and immunotherapy resistance.

Alternative splicing is a mode of transcriptional reprogramming and can change the stability, transport, and translation efficiency of mRNA through different splice forms, thereby directly or indirectly affecting gene regulation [11]. Dysregulation of RNA splicing is generally a hallmark of almost all tumors [12]. Cancer-associated splicing alterations are caused by repetitive mutations and the altered expression of transport factors that control splicing, catalysis, and regulation [13]. Aberrant alternative splicing can promote tumorigenesis through various mechanisms, including increased cell proliferation, decreased apoptosis, increased migration and metastatic potential, resistance to chemotherapy, and immune surveillance evasion [14, 15]. The generation of new abnormal proteins in the context of splicing events may render these cells vulnerable to pharmacological and immunological drugs that target these proteins or their associated pathways [16–18].

The evolution of high-throughput analysis techniques and the increasing availability of transcriptome data have led to an increased number of detected splicing variants and aberrant splicing events [19, 20]. However, how alternative splicing regulates drug resistance in targeted therapy and immunotherapy in melanoma is unclear. This review summarizes and discusses the mechanism of alternative splicing in the pathogenesis and progression of melanoma to address the current clinical dilemma of targeted therapy and immunotherapy resistance.

### Altered expression of splicing factors in melanoma

Splicing factors participate in life activities throughout the body and act like “scissors” to accurately repair pre-mRNA, remove redundant parts, form multiple mRNA sequences, and translate them into protein isomers with different biological functions [21]. The pre-mRNA splicing pathway is a complex cycle involving the assembly, activation, splicing, and depolymerization of multiple RNA and protein components. Each splicing cycle comprises two consecutive transesterification steps. In the first step, the 5′-exon is released, forming an intron chain intermediate, often referred to as the branching process. The second step is exon ligation, where the 5′-exon is interconnected with the 3′-exon [22]. Splicing factors facilitate spliceosome splicing by stabilizing the active site and forming part of the dynamic spliceosome complex.

Alterations in splicing factors are associated with tumor development and progression in various cancer types [13, 14, 23]. The following sections describe the presence and roles of several splicing factors from different protein families in melanomas.

#### Serine/arginine-rich proteins

Most serine/arginine-rich (SR) proteins are splicing activators in tumor pathology [24]. They bind to the pre-mRNA of the exon splicing enhancer and enable exon recognition through spliceosomes, facilitating exon inclusion [25–27]. SR proteins interact with other spliceosomal components via the SR structural domain, linking the 5′- and 3′-splice site binding components, U1 small nuclear ribonucleoproteins complex (U1 snRNP) and U2AF to trigger U1 snRNP, the combination of pre-mRNA containing the 5′-splice site (Table 1) [28, 29].

**Table 1:** SR family

	Targeted Gene	Effect
SRSF1	circMYC	Affect lactate dehydrogenase activity
SRSF2	/	T cell maturation
SRSF3	MDM4v6	p53-MDM4 axis
SRSF6	BIM	Inhibit apoptosis pathway
SRPK1	VEGF	Immunological susceptibility
SRPK2	/	Immunological susceptibility

SR splicing factor 1 (SRSF1) is an oncoprotein that positively regulates circMYC expression, potentially affecting melanoma cells [30]. Its phosphorylated isoform, SRPK1, controls pre-mRNA splicing by regulating pro-angiogenic isoforms [31]. In addition, an increased number of immune cells were observed in biopsies of mice treated with SRPK1/2 pharmacological inhibitors of metastatic melanoma [32–34]. In vitro assays indicated that inhibitors increase immunological sensitivity by intensifying the expression of antigen-presenting major histocompatibility complex (MHC) I and MHC II molecules and splenocyte recruitment [34]. This revealed that the antimetastatic effects of SRPK1/2 inhibition may also include enhanced immune responses, suggesting a possible additional functional role of SRSF1/2 in tumor biology [32–34].

SRSF3 regulates the p53-mediated process to suppress tumorigenesis [35]. SRSF3 is a critical enhancer of alternative splicing, inhibits melanoma growth, and amplifies sensitivity to MAPK-targeted therapies via the p53-MDM4 axis in different human melanoma cell lines and xenograft mouse models derived from patients with melanoma [36, 37].

SRSF6 is necessary to increase the Bim variant (a proapoptotic member of the BCL-2 family) splicing [38]. Considering that SRSF6 is upregulated, inhibiting it with small interfering RNA using vemurafenib intercepts Bim variant mediation and apoptosis [39]. Therefore, rendering melanoma cells susceptible to BRAF V600E inhibitors is essential.

In summary, the SR family plays a crucial role in melanoma development and progression by promoting and regulating splice variant synthesis and acting as drug induction mediators or regulators. Although SR proteins have been extensively described in cancer, SR protein dysregulation in melanomas is still unclear and requires further investigation.

#### Heterogeneous nuclear ribonucleoproteins

Heterogeneous nuclear ribonucleoproteins (hnRNPs) are a protein superfamily that binds to pre-mRNAs through RNA-binding regions to form complexes and participate in alternative splicing [40, 41]. hnRNPs are strongly associated with the pathogenesis and development of various cancers; high hnRNP expression levels can promote the proliferation, invasion, and metastasis of cancer cells and influence patient prognosis [42–45]. In addition, high hnRNP expression may participate in tumor resistance through damage repair mechanisms (Table 2) [46].

**Table 2:** hnRNPs

	Targeted Gene	Effect
hnRNP I (PTBP1)	CD44v6	T-cell homeostasis
hnRNP A1	Stress-induced antigens	Enhance translation and recognition by T lymphocytes
hnRNP A2B1	\	Apoptosis of melanoma stem cells
hnRNP C	p53	Cis-element in the 5′ coding region of p53 mRNA
hnRNP U	AKT	Cooperates with nuclear actin in transcriptional regulation

The absence of polypyrimidine tract-binding protein 1 (PTBP1; hnRNP I) in dendritic cells can increase MHC II expression and disrupt T cell homeostasis without involving dendritic cell progression [47]. PTBP1 deficiency in dendritic cells can increase antitumor immunity [48, 49] and is also relevant to the CD44v6 variant expression in melanoma brain metastasis [50]. Therefore, PTBP1 is a leading factor in regulating immune responses.

Other hnRNPs also play a major role in alternative splicing and influence tumor development; however, no related studies have been conducted on melanoma treatment. hnRNP A1 combines with and activates the internal ribosomal entry sequence of melanoma stress-induced antigens. Endoplasmic reticulum stress agonists promote hnRNP A1 translocation and enhance stress-induced antigen translation and recognition by T-lymphocytes in melanoma cells [51]. hnRNP A2B1 is upregulated in melanoma stem cells and may act through post-transcriptional regulation to block melanoma stem cell apoptosis [52]. hnRNP C directly binds to the cis-element of the 5′ coding region of p53 mRNA, promoting p53 translation [53]. hnRNP U is a protein chaperone of protein kinase B (AKT) that interacts and cooperates with nuclear actin in transcriptional regulation; however, additional biochemical examination is needed to verify the assemblage of nuclear AKT and hnRNP in the cell system [54].

The influence of hnRNPs on melanoma has not been extensively explored; however, these findings provide a unique direction for melanoma treatment.

#### Splicing factor 3B subunit 1

Splicing factor 3B subunit 1 **(**SF3B1) encodes subunit 1 of the splicing factor 3b protein complex, which is involved in pre-mRNA splicing. Splicing factor 3b forms the U2 snRNP with splicing factor 3a and a 12 S RNA unit [55, 56].

SF3B1 is the most commonly mutated splicing factor, with approximately 15–20% of mutations occurring in uveal melanoma. SF3B1 mutations in cancers are primarily missense mutations, with three mutation hotspots targeting the R625, K666, and K700 codon positions [55, 57]. K700 mutations are common in hematopoietic malignancies [58], whereas R625 mutations are the most frequent in uveal melanoma [59]. However, codon R625 repeat mutations in SF3B1 in uveal melanomas are absent in most cutaneous melanomas [60]. These findings suggest that the pathogenesis of the mutated genes is distinct; therefore, the target hotspots for each disease are different, or diverse disease biology possibly drives the selection of individual mutations. This emphasizes the genetic diversity between cutaneous and uveal melanomas, and the demand for subtype-specific therapeutics.

Mutations in spliceosomal components alter intragenic splicing, causing intron retention or aberrant alternative splicing, disrupting the balance of protein isoforms and regulating cell growth and differentiation [61]. In in vivo studies, mutant SF3B1 stimulates aberrant splicing and represses downstream genes by negatively regulating AKT and nuclear factor kappa B (NF-κB) [62, 63]. In in vitro knock-in models, cell migration, tumorigenesis, and hypersensitivity to AKT kinase inhibitors were driven through coordinated NF-κB and AKT signaling activation [62]. In uveal melanomas with SF3B1 mutations, these splicing patterns induce the formation of tumor-specific immunogenic neoepitopes [64]. Neoepitopes are attractive targets for adjuvant therapy, in which soluble biospecific reagents are used to redirect the activity of effector T cells with antibodies or affinity-matured T-cell receptors to tumor cells expressing neoepitopes [65, 66].

SF3B1 binds to cyclin-dependent kinase 11 and phosphorylates its N-terminal threonine residue to activate the spliceosome [67]. Phosphorylation is critical for the association of SF3B1 with U5 and U6 snRNAs in activated spliceosomes; therefore, inhibiting SF3B1 phosphorylation is a novel direction for tumor therapy.

### Aberrant alternative splicing variants in melanoma

RNA splicing plays a pivotal role in melanoma [16, 68], and multi-omics approaches have pinpointed it as one of the most unregulated pathways in melanoma [69]. Additionally, a close association exists between alternative splicing and melanoma prognosis [70]. Therefore, alternative splicing in melanomas should be examined to develop new strategies for reversing drug resistance. Aberrant expression or variation in specific mRNA splicing variants is related to cancer initiation, progression, aggressiveness, and drug resistance due to alternative splicing of critical genes [15, 17, 18]. The importance of specific SVs in melanomas is summarized in Table 3. Genes encoded by BRAF, neuroblastoma RAS (NRAS), the BCL-2 family, MDM4, and CD44 have been the most studied.

**Table 3:** The presence and role of specific SVs

Transcript	Splicing event	Functional role	Molecular mechanism
BRAF	Skipping of the BRAF V600E exons 4–8	Resistance to vemurafenib	Missing the RAS-binding domain (RBD)
NRAS	Isoform 1 (canonical)	Resistance to vemurafenib Potentially serve as biomarkers for therapeutic response and disease prognosis	Lower activity of MEK and ERK and a level of activity
	Isoform 2 (insert exon3b)		Caused less activity along the MEK/ERK axis and increased activity of AKT
	Isoform 3(skipping of exon 3)		Lower activity of MEK and ERK and a level of activity
	Isoform 4(skipping of exons 3 and 4)		Lower activity of MEK and ERK and a level of activity
	Isoform 5(the fusion of the beginning of exon 2 with the end of exon 5)		Increased the activity of all downstream targets
BCL-2 Family	Bcl-xL(alternative 5’ splice site selection within exon 2)	Confer chemo-resistance	Binding the BH4 domain in the N-terminal
	Mcl-1 L & Mcl-1 S	Induced apoptosis	Targeting Mcl-1 pre-mRNA with Mcl-1 antisense morpholino oligonucleotides resulted in a shift towards Mcl-1 S expression
	BimS, BimL & BimEL	Induced apoptosis of BRAFV600E melanoma	Unknown
MDM4	MDM4-S(skipping of exon 6)	Increased sensitivity to cytotoxic chemotherapy and to inhibitors of the BRAF (V600E) oncogene	A negative regulator of p53
CD44	CD44v8-10	related to melanoma metastasis	Regulated by CD82-U2AF2 axis

#### BRAF

**Fig. 1:**
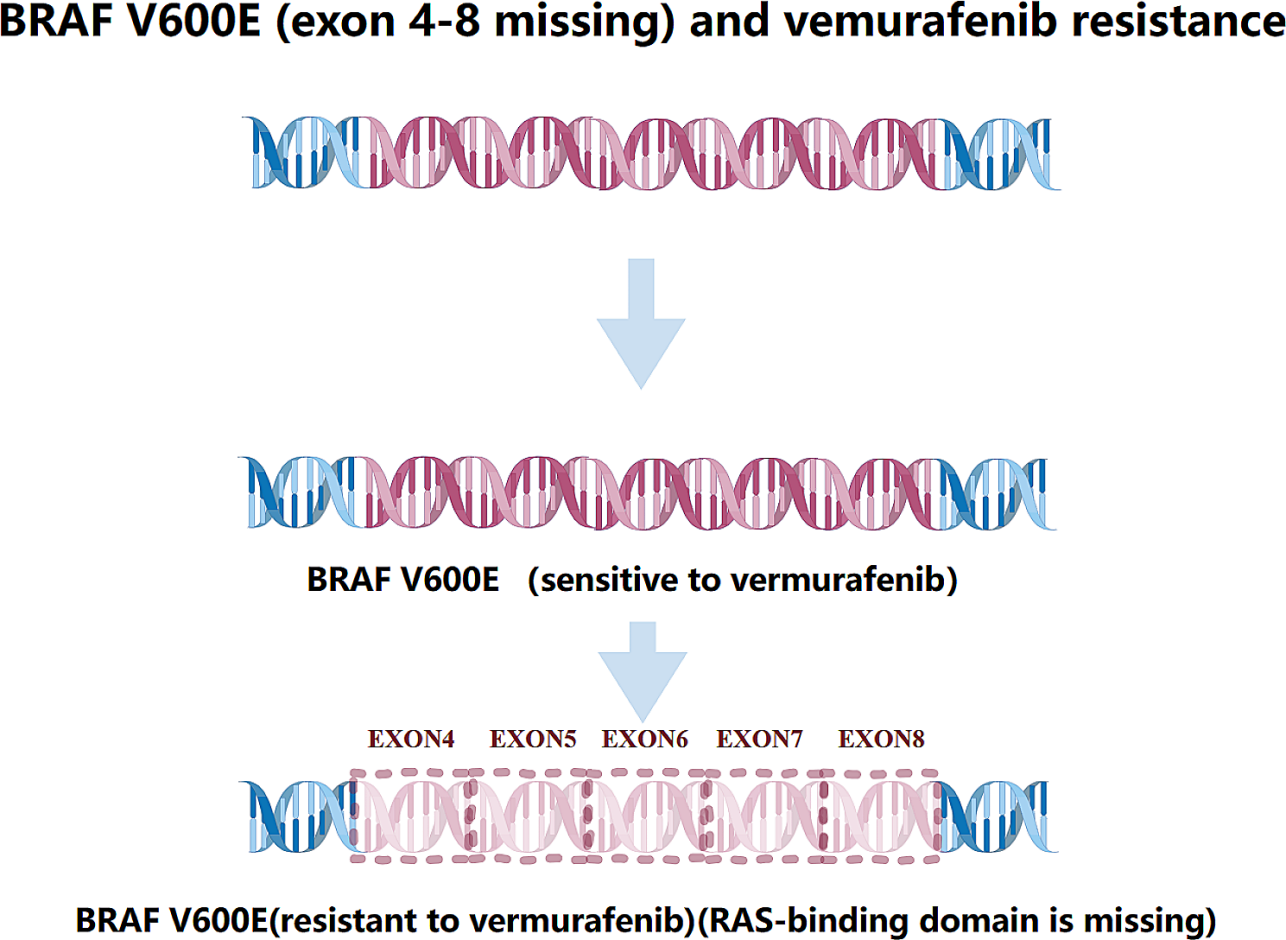
BRAF V600E. This figure schematically depicts the main linear alternative splicing events of BRAF V600E. The RAS-binding domain is missing by skipping exon 4–8, which induces the resistance to vemurafenib

BRAF encodes a serine/threonine kinase that is regulated by the MAP kinase pathway [71]. As a direct RAS effector, BRAF dimerizes to catalyze MEK and extracellular-signal-regulated kinase phosphorylation and activation [72]. Approximately 40–60% of melanomas carry mutations in BRAF [73]. The most common mutation is the valine replacement at codon 600 (V600E) with glutamic acid [74]. Vemurafenib and dabrafenib are BRAF V600E inhibitors approved for treating V600E-mutated melanomas [75]. Although 63–76% of patients with advanced melanoma and BRAF V600E mutations benefited clinically from combination therapy, the median progression-free survival was only approximately 9 months, and 90% of patients experienced resistance within one year [75]. One mechanism of resistance to vemurafenib is a point mutation in intron 8, which leads to exons 4–8 being skipped, thereby eliminating the RAS-binding domain (Fig. 1) [76, 77].

The intricacy and heterogeneity of the pathways associated with the development of resistance to BRAF inhibitors make it challenging to defeat acquired resistance with a “one-size-fits-all” approach. Several studies are currently underway to identify new therapeutic combinations that can restrict or prevent the development of resistance to BRAF inhibitors or overcome already-developed resistance. Most findings indicate that disrupting the BRAF–MEK association during BRAF inhibitor therapy is a probable pharmacological target [73, 76–79]. PLX7904 and its clinical analog, PLX8394, inhibit MEK–ERK1/2 signaling and G1/S cell cycle events, effectively blocking the survival and growth of vemurafenib-resistant cells with diverse BRAF V600E splice variants [79]. These inhibitors are effective in vemurafenib-resistant tumors that express BRAF splice mutations and reduce the homodimerization of splice variants. They are currently undergoing preclinical trials and may be second-line treatment options for patients unresponsive to vemurafenib or dabrafenib.

Two conserved phosphorylated residues exist in RAF regulation: serine 365 (S365) within CR2 and serine 729 (S729) in the BRAF C-terminus [80–82]. The mutation of S729 to a non-phosphorylatable residue reduces the interaction between the BRAF V600E splicing variant and MEK, decreases dimerization or oligomerization, and promotes RAF inhibitor sensitivity [83, 84]. Conversely, the S365 mutation increases BRAF V600E homodimerization [83, 85]. Therefore, the induced S729 site mutation or removal of the S365 site may contribute to the resistance to RAF inhibitors [86]. These outcomes provide evidence for aberrantly spliced forms of BRAF V600E to target resistance.

#### NRAS

NRAS encodes a small GTP-binding protein associated with the cell membrane that links cell surface receptor tyrosine kinases to nuclear transcription factors [87]. NRAS is the second most frequently mutated oncogene in melanoma [88]; however, no effective treatment for NRAS mutations exists [89]. Immunotherapy with programmed cell death protein checkpoint inhibitors, such as nivolumab or pembrolizumab, is the first line of treatment for surgically incurable stage III/IV melanoma with NRAS mutations [90]. However, the efficacy of immunotherapies for treating melanomas with NRAS mutations is contentious [91, 92]. Second-line treatments for melanomas with NRAS mutations include inhibiting the MAPK signaling pathway, MEK, or a combination with other drugs [93, 94]. However, the therapeutic potency of existing drugs against melanomas with NRAS mutations is insufficient [94], highlighting the need to identify novel targets.

**Fig. 2:**
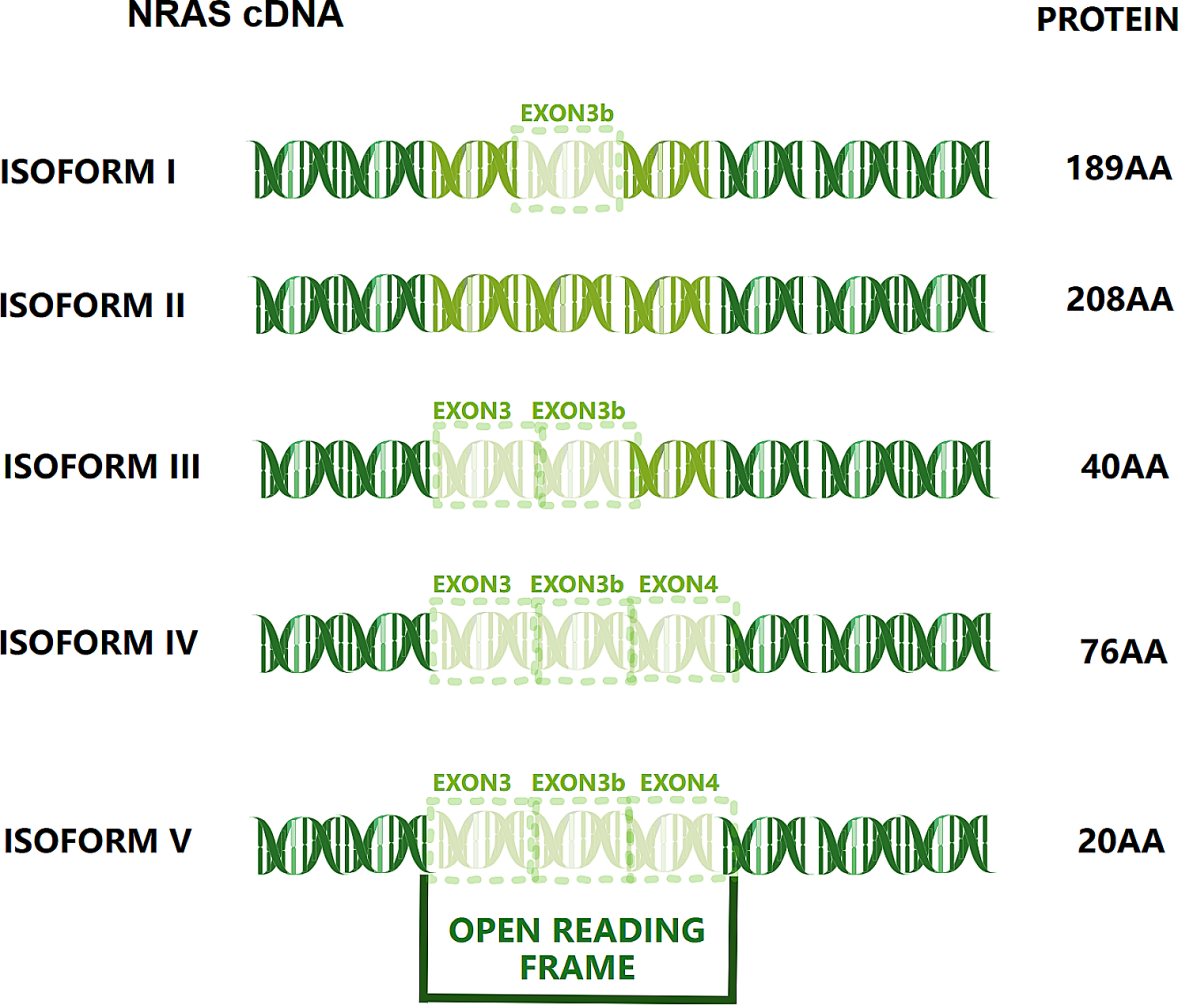
NRAS cDNAs. This figure schematically depicts the five main linear alternative splicing events and protein amino acid length of NRAS. Based on the canonical form 1, the remaining four forms are created by inserting the previously unknown exon 3(b) into form 2, skipped exon 3 into form 3, skipped exons 3 and 4 into form 4, and fused the start of exon 2 with the end of exon 5 into form 5

Since 2014, five NRAS isoforms have been shown to have different expression subtypes, enzymatic activities, and downstream oncogenicity [95]. Based on canonical form 1, the remaining four forms were created by inserting the previously unknown exon 3(b) into form 2, skipping exon 3 into form 3, skipping exons 3 and 4 into form 4, and fusing the start of exon 2 with the end of exon 5 into form 5 (Fig. 2).

Notably, only isoforms 1 and 2 contain codon 61 (exon 3) that can activate constitutive RAS GTPases and switch their conformation toward the GTP-bound active state [96]. The proliferative activity of human melanomas with BRAF V600E mutations increases with NRAS isoform 2 overexpression and concomitant resistance to BRAF inhibitor therapy [97]. Increased PI3K activity in cells expressing isoform 2 is a fundamental mechanism of resistance. Unexpectedly, isoform 5 is localized in the nucleus and lacks GTPase activity, thereby increasing the activity of all downstream target proteins [98] and resistance to vemurafenib [99]. The mechanism of this resistance is unclear; hence, overcoming it requires further exploration. However, in some follow-up samples, the expression of all five NRAS isoforms was detected in the primary tumor and its metastases, which may act as negative prognostic indicators [100].

These new isoforms generate splicing variants that are more immunogenic than a typical protein with a missense mutation encoded by the same gene [99]. Regardless of whether melanoma with NRAS mutations has been deemed hopeless to treat, there is no doubt that novel splice variants inject new energy into targeted therapy or therapeutic resistance [101, 102].

#### BCL-2 family

Members of the BCL-2 protein family control apoptotic pathways [103] and are identified by the appearance of at least one of four BCL-2 homology (BH) domains [104]. This family is divided into a pro-survival and two pro-apoptotic groups. BCL-2, BCL-XL, BCL-W, MCL-1, and BCL2A1 constitute the pro-survival group. The pro-apoptotic subgroups include apoptosis effectors with multi-BH domains (BAX, BAK, and BOK) and apoptosis initiators with mono-BH3 domains (BIM and BAD) [105]. Pro-apoptotic and pro-survival members function through the binding of the BH3 domain to a groove on the surface, which is the switch to apoptosis [106–109].

BCL-XL displays high conformational flexibility with strict regulation of alternative splicing and post-transcriptional induction by transcription factors or microRNAs [110, 111]. Alternative splicing via 5′ splice site selection with exon 2 regulates BCL-XL expression to produce two isoforms [112]. The expression of the MCL splice variant is related to the BRAF mutational status in melanoma cell lines; MCL1L and MCL1S mRNA expression is increased in BRAF V600E mutant melanoma cells [113]. Furthermore, PLX4720 is a selective BRAF inhibitor that upregulates BimS isoform expression to mediate BRAF V600E melanoma cell apoptosis [38]. These findings provide a basis for developing small molecules that directly target BCL-2 proteins in melanoma treatment.

Using BCL-2 family proteins-specific inhibitors is inefficient owing to drug resistance mediated by the overexpression of other BCL-2 proteins. Higher MCL1 and BCL2A1 expression invalidate BCL-2/BCL-XL inhibitors (e.g., ABT199 and ABT263) in clinical and preclinical observations, emphasizing the necessity of associating BCL-2/BCL-XL inhibitors with those of MCL1 or BCL2A1 [114]. In addition, splicing modulators, such as E7107, are ideal combination partners with BCL-2/BCL-XL inhibitors, as they can efficiently modulate MCL1 and BCL2A1 [115]. This combination strategy is under investigation and can effectively inhibit most cancer-related anti-apoptotic BCL-2 family members, thereby expanding to heterogeneous indications and overcoming resistance to current BCL-2/BCL-XL-targeted therapies [116].

These studies show that the BCL-2 family has great latent capacity as a novel approach to cancer treatment. Combination therapies can reverse incomplete responses and treatment resistance to single-agent cancer therapy; however, the development of small molecules that target the BCL-2 family remains challenging.

#### MDM4

**Fig. 3:**
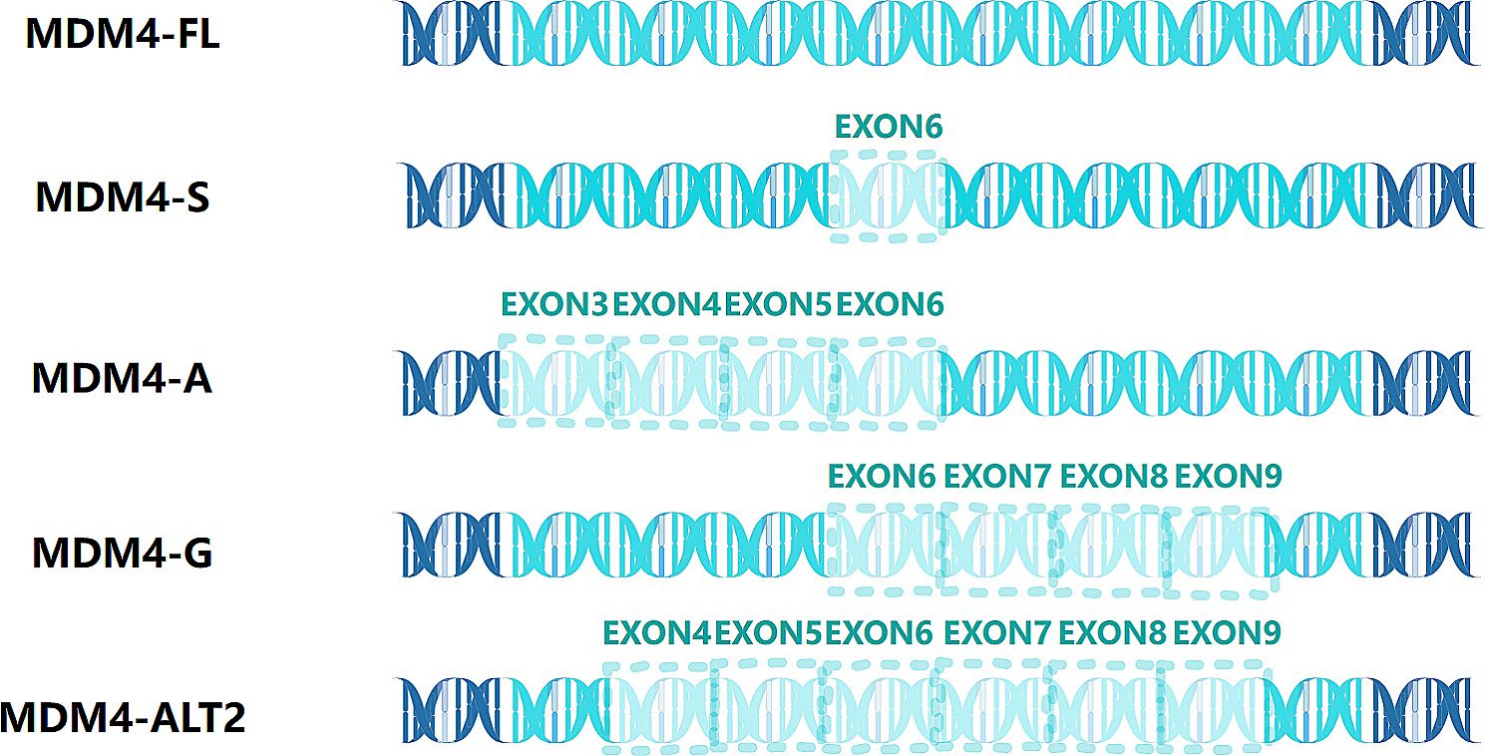
MDM4 splicing events. This figure schematically depicts the main linear alternative splicing events of MDM4. MDM4-FL shows the full exons. Based on MDM4-FL, MDM4-S is produced by skipping exon 6. MDM4-A is by skipping exon 3–6. MDM4-G is by skipping exon6-9. MDM4-ALT2 is by skipping exon4-9

As a critical upstream negative regulator of the tumor suppressor p53 [117], MDM4 is not expressed in most normal tissues but is upregulated in cancer cells to promote overgrowth and inhibit apoptosis [118–121]. Notably, MDM4 exon 6 is skipped in most normal tissues and may act as a switch for the formation of degraded transcription products [122]. This means that the production of the MDM4-S isoform by skipping exon 6 occurs through the nonsense-mediated mRNA degeneration pathway in normal adult tissues. In contrast, the increased inclusion of exon 6 causes the expression of full-length MDM4 in many human cancers [37]. Mechanistically, some SR proteins may be involved in regulating MDM4 splicing variants; however, SRSF3 is one of the most essential enhancers of exon 6 in melanoma cells (Fig. 3) [37, 123].

The selective CDK4/6 inhibitor palbociclib indirectly blocks MDM4 pre-mRNA splicing, thereby reducing its expression and activating p53 [124]. Conversely, inactivating p53 reduces CDK2 inhibition, which replaces CDK4/6 and is a key driver of palbociclib resistance [125, 126]. Thus, inhibiting MDM4-p53 axis regulation can lead to the development of palbociclib resistance [124]. In addition, double targeting effects on CDK4/6 and mutant-BRAF or MEK can regress strong and persistent melanomas with BRAF- and NRAS-mutations in preclinical studies [89, 127–130]. The interaction of MDM4-p53 can promote functional restoration in melanoma cells and sensitize BRAF V600E oncogene inhibitors [131]. However, fluoroquinolones interfere with alternative splicing, causing MDM4 splicing to downregulate MDM4 expression and activate p53 [132].

In summary, MDM4 is a critical factor in p53 functional impairment in human melanoma [131]. Understanding the regulatory mechanism of MDM4 protein levels in cancer is of therapeutic significance. Nevertheless, small molecules or stapled peptides have not been able to selectively and potently disrupt the MDM4-p53 association in clinical trials [37]. However, combining them with fluoroquinolones is a bold new attempt, providing a prospective combination approach that can improve the efficacy of immunotherapy or targeted therapy and reverse resistance.

#### CD44

**Fig. 4:**
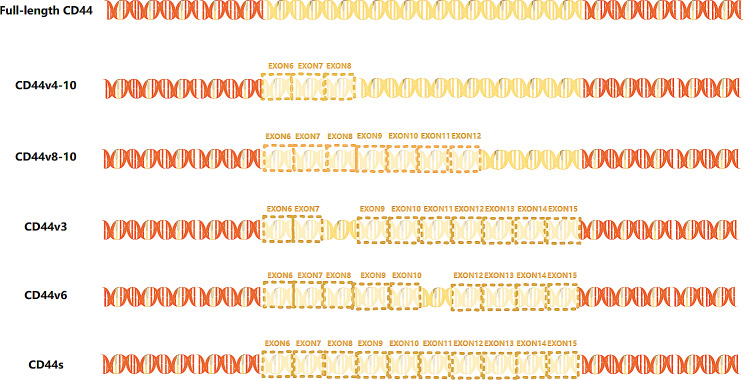
CD44 splicing events. This figure schematically depicts the main linear alternative splicing events of CD44. The full length of the CD44 gene includes 20 exons. The standard CD44 (CD44s) consists of ten constant exons with no variant exons. The variant CD44 (CD44v) has alternative splicing exons deleted or inserted between the N- and C-terminal domains, such as CD44v4-10, CD44v8-10, CD44v3, CD44v6

CD44 is a cell surface glycoprotein involved in cell adhesion and migration [133]. CD44 expression is also upregulated in cancer cell subpopulations and is a molecular hallmark of cancer stem cells [134]. The full-length CD44 gene contains 20 exons. All CD44 family members have homologous domains with exons 1–5 at the N-terminus and exons 16–20 at the C-terminus [135]. CD44 can be divided into two isoforms: standard CD44 (CD44s), which comprises ten constant exons with no variant exons [136], and variant CD44 (CD44v), which has alternatively spliced exons deleted or inserted between the N- and C-terminal domains (Fig. 4) [137].

CD44v is extensively overexpressed in pan-cancer, inducing tumor cell proliferation and drug resistance, hallmarks of cancer stem cells [138]. High expression levels of CD44v6 have been reported in primary melanoma with a propensity for brain metastasis [50]. In primary melanoma, a close correlation exists between splicing factors, such as ESRP1, ESRP2, PTBP1, and U2 snRNP auxiliary factor (U2AF2), and the expression of CD44v6 [50]. In addition, the expression levels of CD44v8-10 and U2AF2 are significantly higher in primary melanoma than in dysplastic nevi and are further increased in metastatic melanoma [139], a crucial milestone during melanoma progression [140]. Mechanistically, U2AF2 facilitates CD44v8-10 alternative splicing in malignant melanoma [139]. In vitro research has provided evidence of the dependence of CD44 expression levels on survival upon vemurafenib treatment [141]. Although animal experiments supported the effect of hyaluronic acid (HA)-modified liposomes on the delivery of chemotherapeutic agents to cancer cells with high CD44 expression, research on targeted therapy and immunotherapy is lacking [142].

Therapeutic approaches include natural selective CD44 inhibition, CD44 decoys, and HA-targeted couples, and these have been studied in different periods of preclinical and clinical trials [143]. Thus, CD44 is a promising therapeutic target for melanoma.

## Discussion

Alternative splicing is a complex cellular mechanism that plays a crucial role in maintaining cell and tissue differentiation and normal cell function [20]. These factors closely regulate splicing events. For example, U2AF2 promotes the alternative splicing of CD44v8-10 in malignant melanoma [139]. Additionally, SRSF3 is a crucial enhancer of MDM4 exon 6 [37, 123]. Furthermore, complex interactions exist between splicing factors; SF3B1 can form spliceosomes with rRNA and inactivate them via phosphorylation [67]. SR proteins and hnRNPs are antagonistic interacting proteins that antagonize the action of hnRNPs in a concentration-dependent manner, preventing exon skipping (164). These complex interactions and tissue-specific roles of splicing factors leave many gaps in research that need to be addressed.

**Fig. 5:**
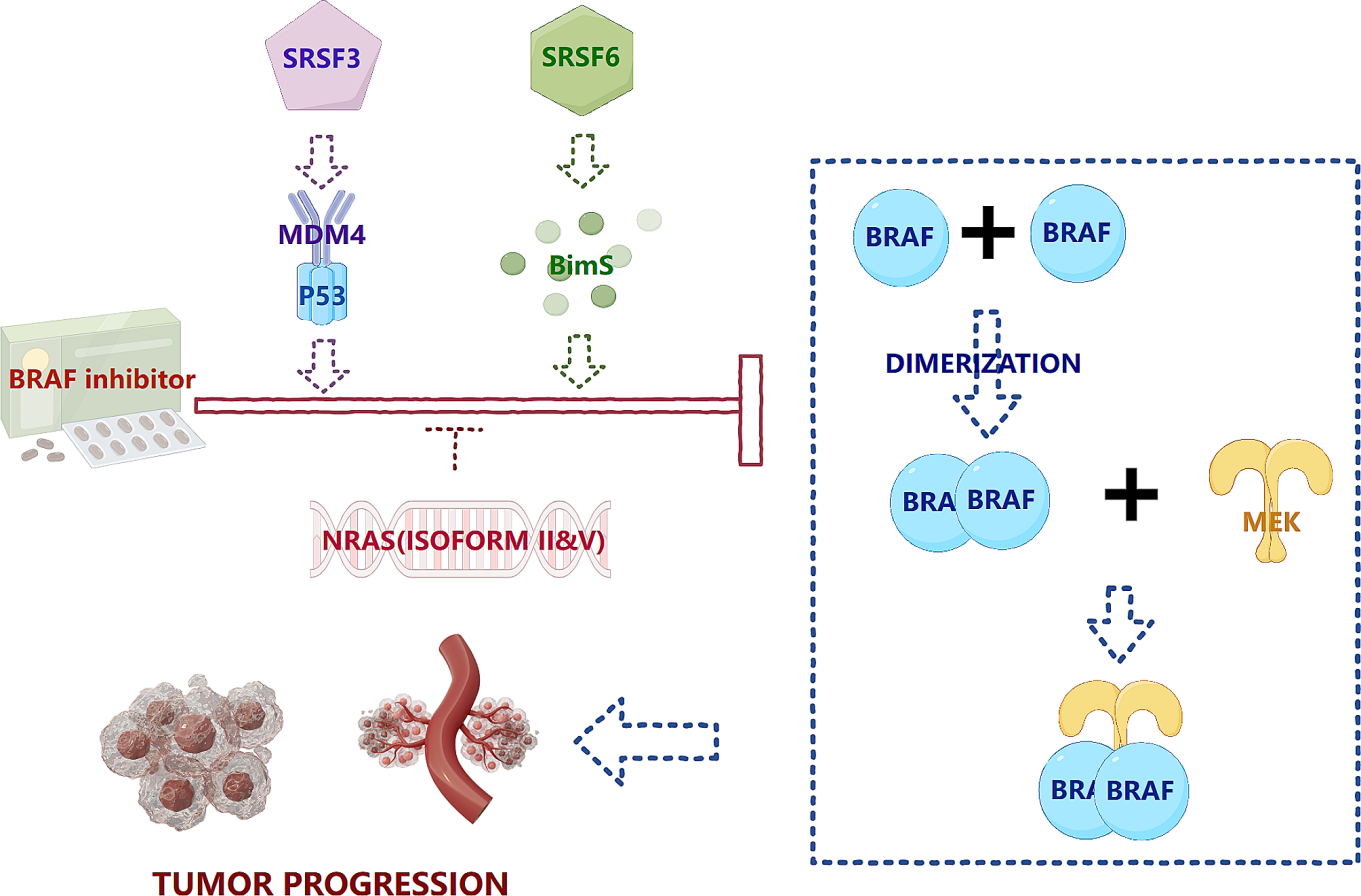
Alternative splicing in targeted therapy resistance. SRSF3 enhances the splicing event of MDM4 to combine with p53. SRSF6 regulates the splicing event of Bim. Both of them can assist BRAF inhibitors to block the process of the dimerization of BRAF and the combination with MEK. Conversely, NRAS increases resistance to BRAF inhibitors through aberrant splicing to generate isoforms II and V

Aberrant alternative splicing is a double-edged sword with completely different effects on various targets. For example, BRAF V600E confers resistance to vemurafenib through exon skipping [76, 77]. In contrast, NRAS increases vemurafenib resistance via aberrant splicing, generating isoforms 2 and 5 [97, 99]. Notably, the BRAF S729 mutation increases sensitivity to RAF inhibitors [86]. Apoptosis in BRAF V600E melanoma cells can be mediated by upregulating BimS isoform expression [38]. However, MDM4 upregulation sensitizes BRAF V600E oncogenic inhibitors [131]. Therefore, individualized evaluation and the design of targeted therapy and immunotherapy must be strictly followed (Fig. 5).

**Fig. 6:**
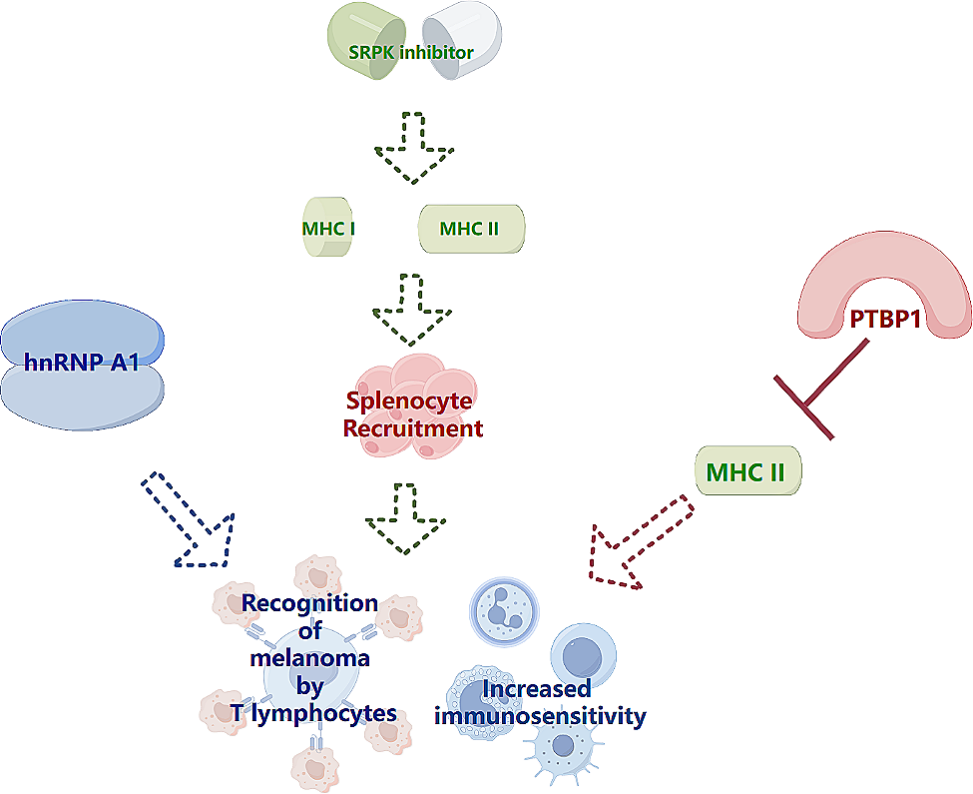
Alternative splicing in targeted therapy resistance. SRPK inhibitors enhance immune sensitivity by enhancing the expression of antigen-presenting MHCI and MHCII molecules and the recruitment of splenocytes. The deletion of PTBP1 can enhance MHC II expression and disrupt T cell homeostasis, and hnRNP A1 assists T lymphocyte recognition of melanoma cells

Most researchers expect to limit, prevent, or overcome targeted therapy and immunotherapy resistance in the form of combination therapy. Oncogenic splicing errors can be alleviated by oligonucleotide-mediated gene therapy (siRNA or SSO), small molecule inhibitors targeting aberrant protein isoforms, and upstream splicing factors [144]. For example, small-molecule inhibitors targeting SF3B1 overcome BRAF V600E-driven vemurafenib resistance by competitively binding to SF3B1, preventing the formation of the U2 snRNP-SF3B1 complex with precursor mRNAs and inhibiting BRAF V600E exon jumping [77, 145]. SRPK is a member of the SR family, whose inhibitors enhance immune sensitivity by enhancing antigen-presenting MHC I and MHC II expression and recruiting splenocytes [34]. Specific deletion of PTBP1 in the hnRNP family in melanoma enhances MHC II expression and disrupts T-cell homeostasis [47], and hnRNP A1 assists in T-lymphocyte recognition in melanoma cells [51]. In addition, oligonucleotide-based therapy is an effective strategy for targeting wild-type or aberrant splicing variants with high selectivity or specificity. SSO has been used to modify MDM4 and BCL2L1 splicing (Fig. 6) [37, 146]. In summary, combination therapies can support the reversal of incomplete responses and treatment resistance in single-dose cancer therapies; however, the complexity and heterogeneity of the pathways involved in their development prevent them from reaching the standard for clinical use, which remains an urgent challenge.

Alternative splice-derived neoepitopes may also serve as potential therapeutic targets [65, 66]. NRAS produces splice variants that are more immunogenic than canonical proteins encoded by the same gene with missense mutations [99], and the number of CD44 variants is further increased in metastatic melanoma [50]. However, many barriers still exist to implementing therapeutic strategies that specifically target these antigens. First, determining whether neoantigens are tumor-specific is crucial. Second, analyzing whether alternative splicing events within a tumor occur elsewhere in the body and not only in the healthy tissue surrounding the tumor is essential [21]. Furthermore, identifying alternative splicing events at the subclonal level in tumors is challenging [147]. These findings indicate the great potential of splice variants in melanoma-targeted therapy and immunotherapy.

In conclusion, this review is the first to summarize the splicing process in melanoma and the changes occurring in this pathway. Alternative splicing is associated with resistance to immunotherapy and targeted therapy in melanomas. With the continuous improvement in science and technology, an in-depth study of the molecular mechanism of alternative splicing in melanoma and continuous exploration of potential novel therapeutic targets can lead to newer and better treatment options for patients with drug resistance.

## Data Availability

Not available.

## Abbreviations

AKT: protein kinase B
BH: BCL-2 homology
CD44s: standard CD44
CD44v: variant CD44
HA: hyaluronic acid
hnRNPs: heterogeneous nuclear ribonucleoproteins
MHC: major histocompatibility complex
NF-κB: nuclear factor kappa B
NRAS: neuroblastoma RAS
PTBP1: polypyrimidine tract-binding protein 1
SF3B1: splicing factor 3B subunit 1
SR: serine/arginine-rich
SRSF1: SR splicing factor 1
SSO: Semi-Synthetic Organism
U1 snRNP: U1 small nuclear ribonucleoproteins complex
U2AF2: U2 snRNP auxiliary factor
V600E: valine replacement at codon 600
